# Polymorphisms but Not Mutations of the *KCNQ1* Gene Are Associated with Lone Atrial Fibrillation in the Chinese Han Population

**DOI:** 10.1155/2013/373454

**Published:** 2013-04-18

**Authors:** Hui-min Chu, Ming-jun Feng, Yi-gang Li, Yi-xin Zhang, Ji-fang Ma, Bin He, Yi-bo Yu, Jing Liu, Xiao-min Chen

**Affiliations:** ^1^Department of Cardiology, Ningbo No. 1 Hospital Affiliated to Medical College of Ningbo University, 59 Liuting Street, Haishu District, Ningbo 315211, China; ^2^Department of Cardiology, Xinhua Hospital Affiliated to Shanghai Jiaotong University School of Medicine, 227 Chongqing Southern Road, Shanghai 200092, China; ^3^Department of Human Population Genetics, Institute of Molecular Medicine, Peking University, 5 Yiheyuan Road, Beijing 100871, China

## Abstract

*Background*. Recent studies suggest that mutation of the slow delayed rectifier potassium channel (IKs) contributes to familial atrial fibrillation (FAF). In the current study, we identified common genetic variants of *KCNQ1* and explored the potential association between *KCNQ1* polymorphism with lone AF (LAF). *Methods*. Clinical data and blood samples were collected from 190 Han Chinese patients with sporadic AF and matched healthy controls. Variants of the *KCNQ1* gene were identified using single-strand conformational polymorphism (SSCP) analysis. A case-control association study in *KCNQ1* identified six known single-nucleotide polymorphisms (SNPs) during SSCP screening of the 190 LAF patients and 190 healthy controls. *Results*. One of the SNPs in *KCNQ1* was strongly associated with LAF; significant allelic association was detected rs59233444 (*P* = 0.013, OR = 1.469, 95% confidence interval (CI): 1.083–1.993). A multiple regression analysis indicated that rs59233444 is an independent risk factor for LAF. Twelve new variants were identified in *KCNQ1*, including one in the 5′-UTR, two in the 3′-UTR, six in introns, two synonymous substitutions, and one missense substitution. Variants c.1009C>T, c.1860C>T, and c.+2285C>T were not present in the 190 controls, and the others were identified in controls at various frequencies. *Conclusions*. rs59233444, a common SNP but not mutation in the coding regions of the *KCNQ1* gene, is a risk factor for LAF in Chinese Han population.

## 1. Background

Atrial fibrillation (AF), a common type of cardiac arrhythmia, is a major risk factor for stroke, heart failure, and other cardiovascular morbidities [1, 2]. In 15%–30% of the AF patients, no underlying heart disease could be identified; these cases are referred to as lone AF (LAF). In about 5% of the cases, family history could be clearly established; these are known as familial AF (FAF). Genetic defect was first reported in Chinese kindred in 2003 [3]. A mutation (S140G) was found in the first transmembrane spanning domain of the cardiac slow delayed rectifier potassium channel (IKs), encoded by the *KCNQ1* gene [4]. Functional analysis of the mutation revealed a “gain-of-function” effect on both KCNQ1-KCNE1 and KCNQ1-KCNE2 ion channels. Since then, “gain-of-function” mutations in other genes encoding potassium ion channels have been found to be associated with FAF, including *KCNE2* [5], *KCNE3* [6], *KCNA5* [7], and *KCNJ2* [8]. Q147R [9], R231C [10], and S209P [11] mutations of the *KCNQ1* gene were also reported.

Interestingly, the gain-of-function mutation of KCNE5 is also linked to LAF [12]. The *KCNE5 *gene product MiRP4 suppresses the IKs current and downregulates the *β*-subunit of the KCNQ1. The mutant KCNE5 L65F fails to suppress IKs, yielding a current indistinguishable from that recorded in the absence of KCNE5. There is evidence of a heritable contribution to LAF, where a polymorphism (S38G) in Mink (KCNE1) was associated with nonfamilial AF [13] (the sporadic LAF). These results indicate that a polymorphism (S38G) and a mutation (S140G) in different genes (*KCNE1* and *KCNQ1*, resp.) encoding different subunits of the same ionic channel (IKs) may be responsible for the development of nonfamilial and familial AF. These results also suggest that familial and nonfamilial AF (sporadic LAF) may share a common pathological mechanism and provide justification to test *KCNQ1* as a candidate gene for LAF.

In the current study, we compared *KCNQ1 *variants in 190 Chinese Han patients with LAF and 190 healthy controls. We also performed a case-control association study for several common SNPs in* KCNQ1*.

## 2. Methods

### 2.1. Study Subjects

Consecutive patients with LAF referred to the cardiology department of Ningbo No. 1 Hospital and Shanghai Xinhua Hospital from June 1, 2007 to September 27, 2009 were enrolled. AF was defined as replacement of the sinus P waves by rapid oscillations of fibrillation waves that varied in size, shape, and timing and were associated with an irregular ventricular response when atrioventricular conduction was intact. LAF was defined as AF occurring in patients <60 years of age without identifiable causes, including hypertension, overt structural heart disease, or thyroid dysfunction. FAF was defined as the presence of LAF in one or more first-degree relative of the indexed case. Each patient underwent a physical examination and a standardized interview to identify past medical conditions, medications, symptoms, family history, and possible triggers for the initiation of AF. All patients were evaluated by 12-lead electrocardiogram (ECG), echocardiogram, and laboratory studies.

Normal control individuals were selected from a cross-sectional, population-based cohort of 190 individuals from Chinese Han people in Southern China. Each subject underwent a comprehensive medical evaluation consisting of a medical history, a physical examination, echocardiography, and electrocardiography. We selected age-, gender-, and ethnicity-matched controls for our study from this population cohort. Control subjects did not have a history of or clinical evidence for AF or any structural disease. Genomic DNA was isolated from peripheral blood leukocytes using standard protocols with the Wizard Genomic DNA Purification Kit (Agilent). This study was approved by the Institution of Ningbo Medical Societies, and all patients gave written informed consent.

### 2.2. Mutation Analysis by SSCP and DNA Sequencing

Exons and exon-intron boundaries of the *KCNQ1* gene were amplified by PCR using standard conditions with primers designed from the published *KCNQ1* sequences in the NCBI database (Accession number: NG_008935.1). PCR was performed in a 25 *μ*L volume containing 200 pmol of each primer, 10 ng of genomic DNA, 2.5 *μ*L of 10 × PCR buffer with 1.5 mmol MgCl_2_, 100 *μ*mol deoxynucleotide triphosphates, and 1 unit of Taq DNA polymerase (Solarbio).

Amplified samples were diluted twofold with 6 *μ*L of formamide buffer (90% formamide, 1 mmol EDTA, 0.2% bromophenol blue, and 0.1% xylene cyanol). The mixture was denatured at 96°C for 3 minutes, then cooled rapidly on ice, and held for 5 minutes. For each sample, 7 *μ*L was loaded onto 10% nondenaturing polyacrylamide gels (acrylamide to bisacrylamide ratio = 40 : 10) and electrophoresed at 80 V for one half hour to two hours at room temperature. The gel was stained with 0.1% silver nitrate and visualized with a 2% NaOH solution (containing 0.1% formaldehyde). Aberrant conformers were directly sequenced with ABI 3130XL instruments (Applied Biosystem), and the sequence was analyzed with Sequence Scanner Software (Version 1.0).

### 2.3. SNP Genotyping

Subsequently, a case-control association study was performed with known SNPs, which were identified from mutation screening. Six SNPs (rs59233444, rs1057128, rs163150, rs760419, rs163160, and rs2075870) were genotyped using direct DNA sequencing (ABI 3130XL, Applied Biosystems). The PCR products were sequenced using forward and/or reverse PCR primers.

### 2.4. Statistical Analysis

Hardy-Weinberg equilibrium calculations were applied to analyze the distribution of genotypes. A *χ* ^2^ test was used to compare allele and genotype frequencies between the cases and controls and to obtain odds ratios (ORs) with 95% confidence intervals (CIs). The SPSS statistical software (Version 18.0) was used for analyzing LD, and haplotypes were calculated using the Haploview software package. Statistical differences were judged significant at *P* < 0.05. The multivariate logistic analysis included age, gender, diabetes, drinking, and smoking habits as covariates.

## 3. Results

### 3.1. Characteristics of the Study Population

A total of 190 patients with LAF and 190 controls were enrolled for the study. Among the 190 patients with LAF, 3 had at least one first-degree relative with AF. The clinical characteristics of these 190 patients are summarized in Table 1. No significant differences were seen between the case patients and control subjects with regard to age, sex, diabetes, smoking and drinking habits, left ventricle ejection fraction, left ventricular end-diastolic diameter, and left ventricular end-systolic diameter. However, the left atrial dimension in the case patient cohort is larger than that in the control cohort (38.8 ± 6.6 mm versus 35.4 ± 4.8, *P* < 0.01). All subjects in our study were of Han ethnic origin.

### 3.2. Identification of Variants in LAF

**Figure 1:**
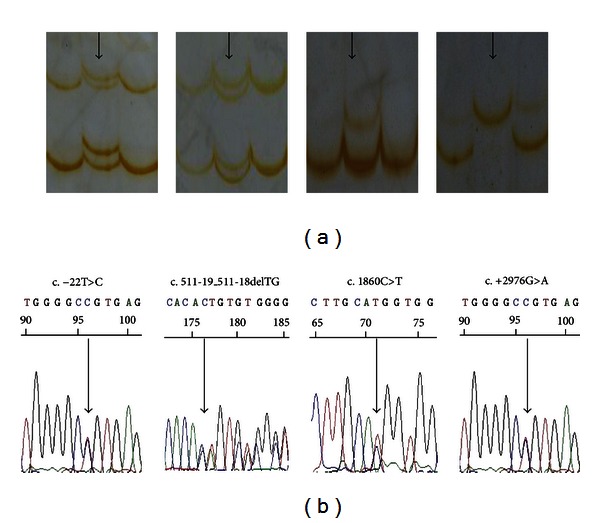
A representative image of an aberrant single-strand conformation found in the LAF using the single-strand conformation polymorphism (SSCP) procedure (a) and confirmed with direct DNA sequencing (b).

To identify mutations or rare polymorphisms associated with AF, all exons and exon-intron boundaries of *KCNQ1* were screened by SSCP analysis. PCR products of aberrant conformers were directly sequenced to identify polymorphisms. A representative portion of the aberrant conformers found in LAF by SSCP and DNA sequencing is shown in Figure 1. A total of 12 variants were identified by our analysis (Table 2). One variant at the 5′-UTR, c.−22T>C, was detected in 2 patients and 1 control, indicating that this variant is a rare polymorphism. Six variants were found in the exon-intron boundaries, including c.511-19_511 18delTG, c.1128+3G>A, c.1590+31A>T, c.1684+23G>A, c.1685−43G>A, and c.1795−18C>T. We analyzed these six intronic variants with Human Splicing Finder (version 2.4.1) and found that they were unlikely to affect a splicing site. Three variants were identified in the coding region of *KCNQ1*, including two synonymous mutations, c.1009C>T and c.1860C>T, that were only detected in three total patients and were not found in the controls, suggesting that they are potentially mutations. The other missense change was identified in exon16, c.1945G>A, and results in a D to N substitution at amino acid 649. This appears to be a polymorphism that is not associated with LAF, because it was present in four of the cases and five of the controls. Two variants were identified in the 3′-UTR, one of which, c.+2285C>T, was found in one patient out of 190 cases and in none of the 190 controls. The other variant, c.+2976G>A, was found in five of 190 LAF patients and in three of the 190 controls. We analyzed these two variants with Patrocles Finder (http://www.patrocles.org/) and did not find evidence of microRNA binding; therefore, they are likely rare nonfunctional polymorphisms. In addition to the 12 novel variants that we reported above, we found 12 known polymorphisms with different frequencies in the cohort of 190 LAF patients (Table 3).

### 3.3. Association of SNPs in *KCNQ1* with LAF

Mutation analysis of all translated *KCNQ1* exons in 190 LAF patients did not reveal a single mutation that clearly altered the splice junction or changed amino acid polarity. Therefore, we focused on six previously characterized SNPs that we identified in our cohort from gene mutation sequencing. Genotyping for six SNPs was done through direct DNA sequencing of rs59233444, rs1057128, rs163150, rs760419, rs163160, and rs2075870. The genotypic frequencies of the six SNPs in the controls were not significantly different from Hardy-Weinberg equilibrium. One of the six SNPs was associated with LAF in the additive and dominant genetic patterns (Table 4). For rs59233444, the genotype distribution is significantly different between (*—*/*—*, GG*—*, GG/GG) LAF patients and controls (49.5, 44.2, and 6.3% versus 34.7, 56.8, and 8.5%, *P* = 0.014). An allelic association with LAF was found by both [2] analysis and a regression test (Table 5). The GG minor allele frequency was 36.8% in the LAF group, compared with 28.4% in the normal controls (OR 1.469, 95% CI: 1.083–1.993, *P* = 0.013), and the A minor allele frequency was 23.4% in the LAF patients, compared with 16.3% in the normal controls (OR 1.885, 95% CI: 1.328–2.676, *P* < 0.01) (Table 6).

In this study, we found rs59233444 were the risk factors for LAF. Moreover, we analyzed all confounding factors for LAF including sex, age, smoking, drinking and hypertension using multiple regression analysis. We found that rs59233444 was the independent risk factor for LAF excluding other risk factors (Table 7). We performed a linkage disequilibrium test and demonstrated a low LD for rs59233444 (D′ = 0.342) (data not shown).

## 4. Discussion

*KCNQ1 (KVLOT1)* channel subunits coassemble with KCNE1 (Mink) subunits to form channels that conduct the slow delayed rectifier K^+^ current, *IK*_*s*_ in the heart which is important for normal termination of the plateau phase, and repolarization of atrial and ventricular action potentials. Mutations in *KCNQ1* were first identified as being the molecular basis of autosomal dominant atrial fibrillation in a single family from China in 2003 [3]. Subsequent studies have implicated potassium ion channel mutations in the pathogenesis of AF [3, 5–8, 10–12]. Although the study of rare familial forms of atrial fibrillation provides insight into the molecular pathways involved in selective cases of the disease, these genetic defects may not be representative of the pathogenesis in the more common, nonfamilial forms found in sporadic AF patients. Therefore, we focused on whether the *KCNQ1* gene was associated with LAF and screened for *KCNQ1* mutations in a cohort of 190 unrelated individuals with LAF.

Despite a plausible rationale for *KCNQ1* as a candidate gene for LAF, we did not identify any *KCNQ1* mutations in our cohort of 190 patients with sporadic LAF. There are several possible explanations for this. First, although AF has an inheritance tendency recognized by many studies, most of the gene mutations found in AF are those that are associated with a family history [3, 6–8, 10–12, 14–18]. Patients with sporadic AF are more likely to be associated with functional polymorphisms rather than mutations [19–23]. Second, AF is a genetically heterogeneous disorder, so a large sample is generally required to sufficiently screen for *KCNQ1* mutations that cause LAF. Finally, certain types of *KCNQ1* mutations would be missed by our SSCP methodology owing to this method's <100% sensitivity. Despite these limitations, our results are consistent with other published results [24].

In addition to the 12 variants in *KCNQ1* that we identified in LAF cases, we uncovered 12 known SNPs from LAF groups at different frequencies. After initial comparison of each of the 12 SNP frequencies that we obtained from the case group with the Han Chinese population in HapMap, six of twelve SNPs were targeted for the case-control association study. Finally, one of the six SNPs was determined to be associated with LAF which located in the *KCNQ1* intron2. The GG minor allele conferred an odds ratio (OR) for developing LAF of 1.469 (95% CI: 1.083–1.993, *P* = 0.013), and the A minor allele conferred a higher OR of 1.885 (95% CI: 1.328–2.676, *P* < 0.01). After correcting for the confounding factors, rs59233444 was found to be a risk factor for LAF. Given the incidence of AF, it would be of importance to determine the functional relevance of the one SNP. It is formally possible that the association described here is attributable to LD between *KCNQ1* gene polymorphisms or to another nearby susceptibility gene.

## 5. Conclusions

Mutations in the coding regions of *KCNQ1* are not a common cause for LAF. Several SNPs were identified in coding and noncoding regions of *KCNQ1*. One SNP in *KCNQ1* (rs59233444) is associated with LAF. Rs59233444 can be deemed as a risk factor for being susceptible to AF in Han Chinese people.

## Figures and Tables

**Table 1:** Clinical characteristics of the study population.

	Case (*n* = 190)	Control (*n* = 190)	*P*
Age^a^ (years, mean ± SD)	55.4 ± 6.3	55.2 ± 7.6	0.211
Gender (female, *n* (%))	68 (35.8%)	71 (37.4)	0.831
Diabetes^b^ (*n* (%))	8 (4.2%)	10 (5.3%)	0.810
Smoking habit (*n* (%))	60 (31.6%)	54 (28.4%)	0.576
Drinking habit (*n* (%))	55 (33.7%)	43 (22.6%)	0.197
Family history	3 (1.6%)	NA	NA
Left atrial dimension (mm)	38.8 ± 6.6	35.4 ± 4.8	<0.01
Left ventricle ejection fraction (%)	64.8 ± 8.9	66.1 ± 9.1	0.215
LVEDD (mm)	48.9 ± 4.9	48.9 ± 7.2	0.542
LVESD (mm)	31.3 ± 4.2	31.1 ± 5.6	0.253

NA: data not available.

^a^Age was defined as the age at the sample collection.

^b^Diabetes was defined as ongoing therapy of diabetes or a fasting plasma glucose level of ≥7.0 mmol.

**Table 2:** List of all *KCNQ1* variants identified in the present study.

Variation	Amino acid change	Frequency in patients	Conserved	Frequency in controls
c.−22T>C	—	2/190	No	1/190
c.51119_51118delTG	—	2/190	No	3/190
c.1009C>T	A336A	1/190	Yes	0/190
c.1128+3G>A	—	2/190	No	1/190
c.1590+31A>T	—	5/190	No	3/190
c.1684+23G>A	—	2/190	No	3/190
c.1685−43G>A	—	1/190	No	2/190
c.1795−18C>T	—	3/190	No	2/190
c.1860C>T	H620H	2/190	Yes	0/190
c.1945G>A	D649N	4/190	No	5/190
c.+2285C>T	—	1/190	Yes	0/190
c.+2976G>A	—	5/190	No	3/190

**Table 3:** Twelve known polymorphisms found in LAF patients.

SNPs	Regions	Minor allele	Amino acid change	Han Chinese MAF
rs1800170	Exon2	T	I145I	0.091
rs59233444	Intron2	GG	—	N.A
rs12786951	Intron6	G	—	0.489
rs12577654	Intron12	T	—	0.057
rs760419	Intron12	G	—	0.318
rs163160	Intron12	G	—	0.244
rs2075870	Intron12	A	—	0.170
rs1057128	Exon13	A	S546S	0.289
rs163150	Intron14	A	—	0.289
rs2519184	3′-UTR	A	—	0.034
rs45510192	3′-UTR	A	—	N.A
rs394656	3′-UTR	C	—	N.A

MAF (minor allele frequency) of Han Chinese is based on HapMap Data; N.A: not available from HapMap Data for Han Chinese population.

**Table 4:** Genotypic association of one SNP in *KCNQ1* with LAF in the Han Chinese population.

SNP	Model	*P*	OR (95% CI)
	Add	0.014	1.579 (1.128–2.210)
rs59233444	Rec	0.432	1.364 (0.627–2.967)
	Dom	0.004	1.840 (1.218–2.779)

Add: additive model; Rec: recessive model; Dom: dominant model; *P*: *P* value from logistic regression; OR (95%): odds ratio with 95% confidence interval.

**Table 5:** Distribution of rs59233444 genotypes in patients and controls.

rs59233444	*N*		Genotype	
		—/—	GG/—	GG/GG
Case	190	66 (34.7%)	108 (56.8%)	16 (8.5%)
Control	190	94 (49.5%)	84 (44.2%)	12 (6.3%)
		*χ* ^2^ = 8.471, *P* = 0.014		

**Table 6:** Allelic association for rs59233444 in LAF.

SNP	Allele (major/minor)	MAF (case/control)	P	OR (95% CI)
Rs59233444	—/GG	0.368/0.284	0.013	1.469 (1.083–1.993)

**Table 7:** Multiple regression analysis for LAF in rs59233444.

Risk factors	*P* value
Sex	0.789
Age	0.530
Smoking	0.689
Drinking	0.744
Hypertension	<0.001
rs59233444	0.005
